# Supplementary material for: Bioinformatic Analysis of the CXCR2 Ligands in Cancer Processes
Source: Int J Mol Sci. 2023 Aug 27;24(17):13287. doi: 10.3390/ijms241713287 (PMC10487711; doi:10.3390/ijms241713287)
Supplement: Supplementary file 1 [file ijms-24-13287-s001.zip › ijms-2558970-supplementary.pdf]

Table S1. The most important miRNAs regulating the expression of CXCR2 ligands.

Ligand CXCR2	Predicted miRNAs		
	**Target Score in the range of 100-90**	**Target Score between 89-85**	**Target Score between 84-80**
CXCL1	miR-532-5p	miR-95-5p, miR-302a-3p, miR-302b-3p, miR-302c-3p, miR-302e, miR-302d-3p, miR-548o-3p, miR-570-3p, miR-1323, miR-5584-3p	miR-372-3p, miR-373-3p, miR-520a-3p, miR-520b-3p, miR-520c-3p, miR-520d-3p, miR-520e-3p
CXCL2	miR-95-5p, , miR-192-5p, miR-215-5p, miR-1266-3p	miR-7-1-3p, miR-7-2-3p, miR-466, miR-495-3p, miR-532-5p, miR-4789-3p, miR-5688, miR-5692a	miR-582-5p, miR-676-5p, miR-3143
CXCL3	let-7c-3p, miR-642a-3p, miR-642b-3p, miR-4291, miR-4753-3p, 5582-3p	miR-194-5p, miR-335-3p, miR-548l, miR-5692a, miR-12136	miR-425-5p, miR-4655-3p, miR-4696
CXCL5	miR-25-3p, miR-32-5p, miR-92a-3p, miR-92b-3p, miR-190a-3p, miR-363-3p, miR-367-3p, miR-548ah-5p, miR-656-3p, miR-1277-5p, miR-3609, miR-4776-3p, miR-5011-5p, miR-5692b, miR-5692c	miR-376c-3p, miR-380-3p, miR-548n, miR-548p, miR-889-3p, miR-3163, miR-4687-5p, miR-4753-3p, miR-6853-3p, miR-6882-5p, miR-7161-5p, miR-8080	miR-33a-3p, miR-330-3p, miR-410-3p, miR-494-3p, miR-548t-5p, miR-548u, miR-548az-5p, miR-651-3p, miR-3160-5p
CXCL6	miR-20a-5p, miR-20b-5p, miR-519d-3p, miR-526b-3p, miR-548t-3p, miR-548aa, miR-548ap-3p, miR-3148, miR-6074	miR-17-5p, miR-93-5p, miR-106a-5p, miR-106b-5p, miR-3123, miR-3606-5p	miR-1178-3p, miR-1224-5p, miR-1272, miR-4477b, miR-6885-3p, miR-7159-3p, miR-8087
PPBP	miR-3140-5p, miR-5009-3p	miR-629-5p, miR-3152-3p, miR-4524a-3p, miR-4795-3p	miR-146a-5p, miR-146b-5p, miR-320a-5p, miR-1267, miR-1343-3p, miR-3668, miR-6783-3p, miR-7153-5p,
CXCL8	miR-140-3p, miR-548at-5p, miR-3671, miR-5692a	miR-153-5p, miR-2115-3p, miR-4312, miR-4436b-5p, miR-4699-3p, miR-4782-5p, miR-5706	miR-376a-2-5p, miR-493-5p, miR-500a-5p, miR-545-5p, miR-607, miR-1294, miR-4687-3p, miR-6732-3p, miR-9986

Table S2. Involvement of CXCR2 ligands in adrenocortical carcinoma (ACC) tumorigenesis.

Name of the cancer	*CXCL1*	*CXCL2*	*CXCL3*	*CXCL5*	*CXCL6*	*PPBP*	*CXCL8*	*CXCR1*	*CXCR2*
Expression level relative to healthy tissue	=	↓	=	=	=	=	=	=	=
Impact of elevated expression on prognosis	=	=	=	=	N/A	↓ p=0.061	↓	=	=
Link to lymph node metastasis status	=	↓	=	=	=	↓ p=0.069	=	=	=
Correlation with proliferation	0.03	0.02	0.08	-0.08	0.02	0.15	0.39	0.11	0.24
Correlation with EMT marker: vimentin	-0.01	0.03	0.08	0.02	0.14	0.09	0.11	0.02	0.07
Correlation with EMT marker: N-cadherin	0.11	0.20	0.11	0.00	0.16	-0.02	0.18	-0.14	-0.10
Correlation with EMT marker: E-cadherin	-0.10	0.02	0.09	0.11	0.02	-0.03	0.14	0.22	0.14
Correlation with T_{reg} count	-0.14	-0.03	0.18	0.13	0.01	-0.23	-0.10	-0.22	0.12
Correlation with neutrophil count	0.13	0.00	0.15	0.15	-0.08	0.22*	0.14	0.14*	0.21*
Correlation with MDSC count	0.16	0.07	0.08	-0.13	0.09	-0.02	0.36	0.15	0.02
Correlation with $CD8^{+}$ T cell count	-0.05	0.00	0.06	0.11	0.21	0.15	-0.04	-0.09	0.06
Correlation with NK cell count	-0.09	-0.22	-0.08	0.10	-0.01	-0.08	-0.06	-0.06	0.08
Correlation with conventional (myeloid) DC count	0.01	0.18	0.16	0.02	0.15	0.21	-0.05	-0.05	0.14
Correlation with plasmacytoid DC count	0.04	0.13	-0.16	-0.29	-0.05	0.12	0.03	0.24	0.19
Correlation with endothelial cell count (marker of angiogenesis)	-0.09	0.03	-0.08	-0.16	-0.17	0.15	-0.26	0.17	0.06
Correlation with macrophage count	-0.08*	0.06*	0.04	-0.04	-0.06	0.21*	0.10	0.09	0.11
Correlation with macrophage M1 count	0.01	0.09	-0.05	-0.18	-0.10	0.10	0.05	0.12	0.15
Correlation with macrophage M2 count	0.00	0.13	0.05	0.18	-0.10	0.20	-0.05	0.00	0.12

↑, red background - expression higher in tumor than in healthy tissue; ↓, blue background - expression lower in tumor than in healthy tissue; ↓, red background - worse prognosis; ↑, blue background – better prognosis; red background - positively correlated with the number of cells; blue background - negatively correlated with the number of cells.

Table S3. Involvement of CXCR2 ligands in bladder urothelial carcinoma (BLCA) tumorigenesis.

Name of the cancer	*CXCL1*	*CXCL2*	*CXCL3*	*CXCL5*	*CXCL6*	*PPBP*	*CXCL8*	*CXCR1*	*CXCR2*
Expression level relative to healthy tissue	=	=	=	=	=	=	=	=	↓
Impact of elevated expression on prognosis	=	=	=	=	=	=	=	=	=
Link to lymph node metastasis status	=	=	=	=	=	=	=	=	??
Correlation with proliferation	0.24	0.19	0.26	0.32	0.19	0.12	0.24	0.05	-0.17
Correlation with EMT marker: vimentin	0.25	0.52	0.41	0.41	0.24	0.20	0.19	0.39	-0.15
Correlation with EMT marker: N-cadherin	0.19	0.30	0.26	0.33	0.25	0.10	0.17	0.22	-0.13
Correlation with EMT marker: E-cadherin	-0.06	-0.18	-0.13	-0.07	0.08	-0.10	0.05	-0.09	0.29
Correlation with T_{reg} count	0.18	0.14	0.18	0.16	0.10	-0.01	0.19	0.12	0.02
Correlation with neutrophil count	0.23	0.18	0.20	0.21	0.19	0.23	0.30	0.24	0.15
Correlation with MDSC count	0.23	0.13	0.20	0.27	0.17	0.18	0.24	-0.05	-0.26
Correlation with $CD8^+$ T cell count	-0.11*	-0.05	-0.09	-0.14*	-0.10	-0.11*	-0.11*	-0.06	0.08*
Correlation with NK cell count	0.02	0.03	0.04	0.01	-0.02	-0.03	-0.01	-0.04	-0.03
Correlation with conventional (myeloid) DC count	0.16	0.22	0.20	0.22	0.08	0.11	0.08	0.13	-0.07
Correlation with plasmacytoid DC count	0.18	0.18	0.18	0.16	0.01	0.02	0.09	0.01	-0.17
Correlation with endothelial cell count (marker of angiogenesis)	-0.20	0.10	-0.06	-0.04	-0.18*	0.03	-0.20	0.17	-0.06
Correlation with macrophage count	0.17	0.23	0.20	0.25	0.06	0.03	0.07	0.00	-0.26
Correlation with macrophage M1 count	0.13	0.25	0.19	0.22	0.02	0.02	0.02*	0.03	-0.31
Correlation with macrophage M2 count	-0.11	0.09	0.06	0.06	-0.12	-0.01	-0.20	-0.04	-0.15*

↑, red background - expression higher in tumor than in healthy tissue; ↓, blue background - expression lower in tumor than in healthy tissue; ↓, red background - worse prognosis; ↑, blue background – better prognosis; red background - positively correlated with the number of cells; blue background - negatively correlated with the number of cells.

Table S4. Involvement of CXCR2 ligands in breast invasive carcinoma (BRCA) tumorigenesis.

Name of the cancer	*CXCL1*	*CXCL2*	*CXCL3*	*CXCL5*	*CXCL6*	*PPBP*	*CXCL8*	*CXCR1*	*CXCR2*
Expression level relative to healthy tissue	↓	↓	↓	=	=	=	=	=	=
Impact of elevated expression on prognosis	↑	↑	↑ p=0.056	↑ p=0.08	↑	N/A	=	=	=
Link to lymph node metastasis status	??	↓	↓	??	??	=	↓	=	=
Correlation with proliferation	0.13	-0.08	0.11	0.23	0.09	0.05	0.26	0.00	0.01
Correlation with EMT marker: vimentin	0.36	0.40	0.39	0.38	0.27	0.23	0.33	0.18	0.31
Correlation with EMT marker: N-cadherin	0.10	-0.01	0.10	0.15	0.04	0.11	0.33	0.09	0.19
Correlation with EMT marker: E-cadherin	-0.23	-0.30	-0.25	-0.13	-0.13	-0.07	-0.05	0.09	0.03
Correlation with T_{reg} count	0.017	-0.01	0.01	0.02	-0.02	-0.05	0.18*	-0.02	0.12
Correlation with neutrophil count	0.12	0.09	0.11	0.09	0.13	0.13	0.09*	0.10*	0.13
Correlation with MDSC count	0.19	0.04	0.14	0.21	0.11	0.05	0.19	-0.19	-0.41
Correlation with $CD8^+$ T cell count	0.12	0.01	0.07	0.06	0.12	-0.04	-0.04	-0.02	0.06
Correlation with NK cell count	-0.02	-0.06	-0.05	-0.04	0.02	-0.02	-0.04	0.00	-0.07
Correlation with conventional (myeloid) DC count	0.29	0.17	0.23	0.24	0.27	0.09	0.16	0.08	0.18
Correlation with plasmacytoid DC count	0.16	-0.05	0.11	0.13	0.10	0.01	0.11	0.00	0.06
Correlation with endothelial cell count (marker of angiogenesis)	-0.08	0.15	-0.05	-0.12	0.00	0.12	-0.12	0.16	0.12
Correlation with macrophage count	0.01	-0.13	0.04	0.06	-0.01	0.07	0.19	0.08*	0.30
Correlation with macrophage M1 count	0.15	-0.02	0.15	0.18	0.11	0.09	0.18	0.01	0.14
Correlation with macrophage M2 count	-0.16	-0.10	-0.13	-0.16	-0.16	0.04	0.02	0.15	0.31

↑, red background - expression higher in tumor than in healthy tissue; ↓, blue background - expression lower in tumor than in healthy tissue; ↓, red background - worse prognosis; ↑, blue background – better prognosis; red background - positively correlated with the number of cells; blue background - negatively correlated with the number of cells.

Table S5. Involvement of CXCR2 ligands in cervical squamous cell carcinoma and endocervical adenocarcinoma (CESC) tumorigenesis.

Name of the cancer	*CXCL1*	*CXCL2*	*CXCL3*	*CXCL5*	*CXCL6*	*PPBP*	*CXCL8*	*CXCR1*	*CXCR2*
Expression level relative to healthy tissue	↑	=	↑	=	=	=	↑	=	=
Impact of elevated expression on prognosis	↓	↓	↓	↓	↓ p=0.056	↓ p=0.088	↓	↓	=
Link to lymph node metastasis status	=	=	=	=	=	=	=	=	=
Correlation with proliferation	0.16	0.03	0.06	0.09	0.19	0.01	0.24	0.10	0.18
Correlation with EMT marker: vimentin	-0.05	0.13	0.05	0.25	0.10	0.06	-0.01	0.11	0.01
Correlation with EMT marker: N-cadherin	-0.03	0.19	0.19	0.36	0.02	0.22	0.06	0.11	-0.13
Correlation with EMT marker: E-cadherin	0.18	0.01	0.02	0.05	0.22	0.06	0.19	0.08	0.14
Correlation with Treg count	-0.02	-0.03	-0.04	0.05	0.16*	-0.01	0.03	-0.06	0.03
Correlation with neutrophil count	0.26	0.19	0.24	0.15	0.22	0.23	0.32	0.38	0.24
Correlation with MDSC count	0.19	0.29	0.26	0.23	0.04	0.17	0.36	0.07	-0.35
Correlation with $CD8^+$ T cell count	-0.17	-0.13*	-0.15*	-0.14*	-0.05	-0.15*	-0.29	-0.14*	0.04
Correlation with NK cell count	0.03	0.03	0.01	0.06	-0.01	0.02	0.07	0.02	0.00
Correlation with conventional (myeloid) DC count	-0.16	-0.13	-0.16	-0.12	-0.07	-0.17	-0.30	-0.10	0.07
Correlation with plasmacytoid DC count	-0.16	-0.07	-0.13	-0.12	-0.10	-0.12	-0.18	-0.03	0.05
Correlation with endothelial cell count (marker of angiogenesis)	-0.03	0.15	0.12*	0.21	-0.03*	0.15	0.00*	0.22	-0.02
Correlation with macrophage count	-0.25	-0.21	-0.29	-0.19	-0.18*	-0.17	-0.33	-0.12*	0.07
Correlation with macrophage M1 count	-0.25	-0.18	-0.27	-0.16	-0.15*	-0.14	-0.31	-0.08	0.04
Correlation with macrophage M2 count	-0.24	-0.20	-0.42	-0.19	-0.18	-0.13	-0.39	-0.11	0.02

↑, red background - expression higher in tumor than in healthy tissue; ↓, blue background - expression lower in tumor than in healthy tissue; ↓, red background - worse prognosis; ↑, blue background – better prognosis; red background - positively correlated with the number of cells; blue background - negatively correlated with the number of cells.

Table S6. Involvement of CXCR2 ligands in cholangiocarcinoma (CHOL) tumorigenesis.

Name of the cancer	*CXCL1*	*CXCL2*	*CXCL3*	*CXCL5*	*CXCL6*	*PPBP*	*CXCL8*	*CXCR1*	*CXCR2*
Expression level relative to healthy tissue	↑	↓	↑	↑	↑	=	↑	=	=
Impact of elevated expression on prognosis	=	=	=	=	=	=	=	=	=
Link to lymph node metastasis status	=	=	=	=	=	=	=	=	=
Correlation with proliferation	0.12	0.01	0.28	0.23	0.06	0.17	0.24	0.30	0.35
Correlation with EMT marker: vimentin	0.18	0.09	0.23	0.26	0.29	0.11	0.24	0.24	0.20
Correlation with EMT marker: N-cadherin	-0.10	-0.05	-0.07	-0.10	-0.02	0.20	0.11	0.00	0.02
Correlation with EMT marker: E-cadherin	-0.06	-0.08	-0.08	-0.17	-0.29	-0.09	0.06	-0.31	-0.21
Correlation with T_{reg} count	-0.20	-0.05	-0.15	-0.29	-0.17	-0.24	-0.15	-0.14	0.00
Correlation with neutrophil count	0.05	-0.22	0.12	-0.08	-0.02	0.32	0.16	0.14	0.12
Correlation with MDSC count	0.38	0.26	0.32	0.44	0.39	0.15	0.34	0.32	0.27
Correlation with $CD8^{+}$ T cell count	-0.28	-0.27	-0.19	-0.13	-0.18	-0.07	-0.37*	-0.32	-0.17
Correlation with NK cell count	-0.01	-0.22	-0.31	-0.05	0.10	-0.48*	-0.18	-0.31	-0.35
Correlation with conventional (myeloid) DC count	0.03	0.06	0.22	-0.09	0.00	0.29	0.07	0.05	0.16
Correlation with plasmacytoid DC count	-0.05	0.03	0.12	0.14	0.09	0.35	0.05	0.20	0.25
Correlation with endothelial cell count (marker of angiogenesis)	0.10	-0.04	-0.05	-0.08	-0.08	0.13	-0.14	-0.05	-0.09
Correlation with macrophage count	-0.18	-0.46	-0.18	-0.22	-0.06	-0.09	-0.09	-0.01	0.12
Correlation with macrophage M1 count	-0.27	-0.27	-0.09	-0.10	-0.14	0.24	-0.08	0.01	0.12
Correlation with macrophage M2 count	-0.31	-0.53	-0.20	-0.33	-0.21	-0.13	-0.24	-0.36*	-0.22

↑, red background - expression higher in tumor than in healthy tissue; ↓, blue background - expression lower in tumor than in healthy tissue; ↓, red background - worse prognosis; ↑, blue background – better prognosis; red background - positively correlated with the number of cells; blue background - negatively correlated with the number of cells.

Table S7. Involvement of CXCR2 ligands in colon adenocarcinoma (COAD) tumorigenesis.

Name of the cancer	*CXCL1*	*CXCL2*	*CXCL3*	*CXCL5*	*CXCL6*	*PPBP*	*CXCL8*	*CXCR1*	*CXCR2*
Expression level relative to healthy tissue	↑	↑	↑	↑	=	=	↑	=	=
Impact of elevated expression on prognosis	=	↑	↑ p=0.094	=	=	=	=	=	=
Link to lymph node metastasis status	=	=	=	=	=	=	=	=	=
Correlation with proliferation	0.14	0.17	0.18	0.18	0.05	0.04	0.04	0.05	0.04
Correlation with EMT marker: vimentin	0.05	-0.07	-0.10	0.27	0.34	0.25	0.40	0.47	0.40
Correlation with EMT marker: N-cadherin	-0.15	-0.21	-0.25	0.11	0.16	0.20	0.22	0.28	0.25
Correlation with EMT marker: E-cadherin	-0.11	-0.04	-0.07	-0.04	-0.01	-0.04	-0.10	0.06	0.13
Correlation with T_{reg} count	0.07	0.07	0.08	0.10	0.14	0.03	0.08	0.17	0.23
Correlation with neutrophil count	0.27	0.25	0.25	0.35	0.36	0.18	0.43	0.58	0.54
Correlation with MDSC count	0.02	0.07	0.06	0.00	-0.10	0.02	-0.09	-0.37	-0.34
Correlation with $CD8^{+}$ T cell count	-0.03	-0.02	-0.02	-0.16	-0.08	-0.22	-0.10	0.04	0.07
Correlation with NK cell count	-0.02	0.00	-0.01	-0.02	-0.07	-0.18*	0.02	-0.08	-0.09
Correlation with conventional (myeloid) DC count	0.15	0.07	0.06	0.25	0.31	0.12	0.44	0.49	0.47
Correlation with plasmacytoid DC count	0.08	0.18	0.06	0.06	0.06	-0.16	0.06	0.09	0.10
Correlation with endothelial cell count (marker of angiogenesis)	0.06	-0.02	-0.08*	0.24	0.25	0.17	0.28	0.42	0.38
Correlation with macrophage count	0.08	-0.03	-0.02	0.21	0.23	0.14	0.35	0.43	0.40
Correlation with macrophage M1 count	0.07	-0.03	-0.04	0.24	0.25	0.18	0.39	0.50	0.44
Correlation with macrophage M2 count	0.00*	-0.06	-0.41	0.03	0.03	0.13*	0.12	0.16	0.12

↑, red background - expression higher in tumor than in healthy tissue; ↓, blue background - expression lower in tumor than in healthy tissue; ↓, red background - worse prognosis; ↑, blue background – better prognosis; red background - positively correlated with the number of cells; blue background - negatively correlated with the number of cells.

Table S8. Involvement of CXCR2 ligands in the tumorigenesis of diffuse large B-cell lymphoma (DLBC).

Name of the cancer	*CXCL1*	*CXCL2*	*CXCL3*	*CXCL5*	*CXCL6*	*PPBP*	*CXCL8*	*CXCR1*	*CXCR2*
Expression level relative to healthy tissue	=	=	=	=	=	↓	↓	↓	↓
Impact of elevated expression on prognosis	=	=	↓	=	↓ p=0.061	=	=	=	=
Link to lymph node metastasis status	N/A	N/A	N/A	N/A	N/A	N/A	N/A	N/A	N/A
Correlation with proliferation	0.10	0.33	0.33	0.12	0.03	0.19	0.26	0.13	0.30
Correlation with EMT marker: vimentin	0.29	0.41	0.49	0.50	0.10	0.08	0.26	0.43	0.64
Correlation with EMT marker: N-cadherin	0.15	0.24	0.30	0.39	0.42	0.06	0.24	0.18	0.28
Correlation with EMT marker: E-cadherin	0.00	0.15	0.23	0.24	-0.03	0.18	-0.09	0.42	0.46
Correlation with T_{reg} count	-0.30	-0.27	-0.13	-0.01	-0.22	-0.17	-0.28	-0.21	0.10
Correlation with neutrophil count	0.38	0.31	0.31	0.27	0.11	-0.03	0.13	0.26	0.13
Correlation with MDSC count	-0.03	0.10	0.00	0.04	0.15	0.38	0.16	0.06	-0.19
Correlation with $CD8^+$ T cell count	-0.05	-0.02	-0.03	-0.13	-0.38*	-0.34*	-0.36*	0.10	0.08
Correlation with NK cell count	0.29	0.39	0.35	0.16	-0.15	0.14	0.24	0.26	0.37*
Correlation with conventional (myeloid) DC count	-0.05	-0.19	-0.09	0.03	0.08	-0.35	-0.13	0.13	0.32
Correlation with plasmacytoid DC count	0.10	0.26	0.09	0.15	-0.22	-0.05	-0.25	-0.08	-0.03
Correlation with endothelial cell count (marker of angiogenesis)	0.19	0.34	0.09	0.24	-0.01	0.41	0.13	0.03	-0.29
Correlation with macrophage count	0.30	0.16	0.22	0.33*	-0.03	-0.06	0.31	0.01	0.22
Correlation with macrophage M1 count	0.38	0.26	0.20*	0.30	-0.01	-0.02	0.39*	0.00	0.19
Correlation with macrophage M2 count	0.11	0.05	0.04	0.21	0.07	-0.02	0.18	-0.06	0.02

↑, red background - expression higher in tumor than in healthy tissue; ↓, blue background - expression lower in tumor than in healthy tissue; ↓, red background - worse prognosis; ↑, blue background – better prognosis; red background - positively correlated with the number of cells; blue background - negatively correlated with the number of cells.

Table S9. Involvement of CXCR2 ligands in esophageal carcinoma (ESCA) tumorigenesis.

Name of the cancer	*CXCL1*	*CXCL2*	*CXCL3*	*CXCL5*	*CXCL6*	*PPBP*	*CXCL8*	*CXCR1*	*CXCR2*
Expression level relative to healthy tissue	↑	↑	↑	↑	↑	=	↑	=	↓
Impact of elevated expression on prognosis	=	=	=	↓ p=0.092	=	=	↓	=	=
Link to lymph node metastasis status	=	=	??	=	=	=	↑	=	=
Correlation with proliferation	0.19	0.16	0.20	0.18	0.13	-0.02	0.17	-0.07	-0.14
Correlation with EMT marker: vimentin	-0.05	0.02	-0.08	0.16	0.16	0.01	0.06	0.09	-0.08
Correlation with EMT marker: N-cadherin	-0.25	-0.17	-0.33	0.05	0.01	-0.17	-0.01	-0.15	-0.10
Correlation with EMT marker: E-cadherin	0.07	-0.04	0.04	-0.03	0.05	0.01	0.05	0.03	0.13
Correlation with T_{reg} count	-0.06	-0.08*	-0.08*	-0.07	0.00	-0.08	0.03	-0.09	0.05
Correlation with neutrophil count	0.31	0.16	0.23	0.19	0.24	0.30	0.29	0.34	0.35
Correlation with MDSC count	-0.01	-0.24	-0.26	0.16	0.10	-0.04	0.15	-0.21	-0.21
Correlation with $CD8^+$ T cell count	-0.13	-0.05	-0.09	-0.15	-0.22*	-0.09	-0.15*	-0.08	-0.05
Correlation with NK cell count	-0.04	0.01	-0.03	-0.14	-0.03	-0.09	-0.07	-0.16*	-0.10
Correlation with conventional (myeloid) DC count	-0.15	-0.12	-0.16	-0.04	-0.12	0.01	0.00	0.01	-0.06
Correlation with plasmacytoid DC count	0.13	0.17	0.19	0.08	0.02	0.04	0.05	0.17	-0.04
Correlation with endothelial cell count (marker of angiogenesis)	0.21	0.37	0.33	0.20	0.17	0.17*	0.01	0.26	0.03
Correlation with macrophage count	-0.11	-0.01	0.03	0.18	-0.06	0.08	0.06	0.12	-0.14*
Correlation with macrophage M1 count	-0.03	0.11	0.09	0.20	0.03	0.09	0.10	0.16	-0.15
Correlation with macrophage M2 count	-0.01*	0.23*	-0.27	0.15	-0.08	0.14	-0.05	0.18*	-0.12

↑, red background - expression higher in tumor than in healthy tissue; ↓, blue background - expression lower in tumor than in healthy tissue; ↓, red background - worse prognosis; ↑, blue background – better prognosis; red background - positively correlated with the number of cells; blue background - negatively correlated with the number of cells.

Table S10. Involvement of CXCR2 ligands in glioblastoma multiforme (GBM) tumorigenesis.

Name of the cancer	*CXCL1*	*CXCL2*	*CXCL3*	*CXCL5*	*CXCL6*	*PPBP*	*CXCL8*	*CXCR1*	*CXCR2*
Expression level relative to healthy tissue	=	↑	↑	=	=	=	↑	=	=
Impact of elevated expression on prognosis	↓	↓	=	↓	↓ p=0.096	=	=	=	=
Link to lymph node metastasis status	N/A	N/A	N/A	N/A	N/A	N/A	N/A	N/A	N/A
Correlation with proliferation	-0.17	-0.26	-0.20	-0.20	-0.19	-0.19	-0.29	-0.04	-0.07
Correlation with EMT marker: vimentin	0.26	0.18	0.36	0.31	0.24	0.23	0.26	0.14	0.15
Correlation with EMT marker: N-cadherin	0.12	0.10	0.16	0.12	0.01	0.10	0.04	0.10	0.17
Correlation with EMT marker: E-cadherin	-0.16	-0.15	-0.25	-0.28	-0.29	-0.19	-0.27	-0.12	-0.05
Correlation with T_{reg} count	0.12	0.12	0.11	0.21	0.10	-0.16	-0.01	0.06	0.08
Correlation with neutrophil count	0.09*	0.07*	0.11*	0.10*	0.12	0.16	0.11	0.17*	0.18
Correlation with MDSC count	0.00	-0.14	-0.03	-0.18	0.05	-0.08	-0.01	-0.16	-0.46
Correlation with $CD8^+$ T cell count	-0.08	-0.17	-0.17	-0.09	-0.07	0.01	-0.20*	-0.15	-0.13
Correlation with NK cell count	-0.06	-0.06	0.00	0.00	-0.06	0.02	-0.04	0.02	-0.07
Correlation with conventional (myeloid) DC count	0.10	-0.05	0.15	0.21	0.16	0.08	0.09	0.03	0.06
Correlation with plasmacytoid DC count	-0.10	0.01	-0.05	-0.01	-0.10	-0.05	-0.18	-0.17	0.05
Correlation with endothelial cell count (marker of angiogenesis)	0.15	-0.10	-0.09	0.00	0.03	0.09	0.09	0.17	0.10
Correlation with macrophage count	0.18	0.16	0.19*	0.30	0.28	0.22	0.24	0.08	0.33
Correlation with macrophage M1 count	0.25*	0.18	0.33*	0.42	0.36	0.29	0.33	0.16	0.30
Correlation with macrophage M2 count	-0.02	-0.05	-0.14	0.00	0.04	-0.03	-0.10	-0.03	0.23

↑, red background - expression higher in tumor than in healthy tissue; ↓, blue background - expression lower in tumor than in healthy tissue; ↓, red background - worse prognosis; ↑, blue background – better prognosis; red background - positively correlated with the number of cells; blue background - negatively correlated with the number of cells.

Table S11. Involvement of CXCR2 ligands in head and neck squamous cell carcinoma (HNSC) tumorigenesis.

Name of the cancer	*CXCL1*	*CXCL2*	*CXCL3*	*CXCL5*	*CXCL6*	*PPBP*	*CXCL8*	*CXCR1*	*CXCR2*
Expression level relative to healthy tissue	↑	=	=	=	=	=	↑	=	↓
Impact of elevated expression on prognosis	↓ p=0.076	=	=	=	=	=	=	=	=
Link to lymph node metastasis status	↓	↓	↓ p=0.082	??	↓	=	↓	↓	↓
Correlation with proliferation	0.09	0.07	0.18	0.14	0.12	-0.09	0.19	0.02	0.02
Correlation with EMT marker: vimentin	0.01	0.25	0.25	0.21	0.13	-0.03	0.02	0.17	-0.07
Correlation with EMT marker: N-cadherin	-0.04	0.14	0.19	0.21	0.09	0.01	0.07	0.04	-0.08
Correlation with EMT marker: E-cadherin	-0.03	-0.09	-0.04	0.19	0.13	0.13	0.17	0.15	0.34
Correlation with T_{reg} count	0.016	0.02	0.05	0.01	-0.07	0.03	0.02	0.11*	0.09*
Correlation with neutrophil count	0.32	0.20	0.19	0.24	0.26	0.32	0.33	0.40	0.43
Correlation with MDSC count	0.14	0.18	0.24	0.23	0.13	0.08	0.25	-0.05	-0.34
Correlation with $CD8^+$ T cell count	-0.10*	-0.09	-0.10	-0.24	-0.17	-0.19	-0.25	-0.01	0.13*
Correlation with NK cell count	-0.02	0.00	0.03	0.03	0.00	0.04*	0.06	-0.02	-0.02
Correlation with conventional (myeloid) DC count	-0.05	0.04	0.05	0.01	-0.02	-0.02	-0.07	0.07	0.10
Correlation with plasmacytoid DC count	0.01	0.01	0.00	-0.24	-0.13	-0.18	-0.20	0.07	0.04
Correlation with endothelial cell count (marker of angiogenesis)	0.02	0.18	0.14	0.27	0.24	0.23	0.09*	0.33	0.13
Correlation with macrophage count	-0.12*	0.02	0.01	-0.03	-0.13*	-0.10	-0.09	0.02	-0.09
Correlation with macrophage M1 count	-0.12*	0.04	0.01	-0.05	-0.11*	-0.12	-0.13*	0.03	-0.11*
Correlation with macrophage M2 count	-0.18	-0.07	-0.13	-0.12	-0.17	-0.09*	-0.20	-0.04	-0.06

↑, red background - expression higher in tumor than in healthy tissue; ↓, blue background - expression lower in tumor than in healthy tissue; ↓, red background - worse prognosis; ↑, blue background – better prognosis; red background - positively correlated with the number of cells; blue background - negatively correlated with the number of cells.

Table S12. Involvement of CXCR2 ligands in kidney chromophobe tumorigenesis (KICH).

Name of the cancer	*CXCL1*	*CXCL2*	*CXCL3*	*CXCL5*	*CXCL6*	*PPBP*	*CXCL8*	*CXCR1*	*CXCR2*
Expression level relative to healthy tissue	↓	↓	=	=	=	=	↓	=	=
Impact of elevated expression on prognosis	=	=	=	↓	=	=	↓	=	=
Link to lymph node metastasis status	=	=	=	??	??	=	=	=	=
Correlation with proliferation	0.28	-0.17	0.15	0.27	0.24	0.09	0.29	-0.04	0.12
Correlation with EMT marker: vimentin	0.43	0.48	0.47	0.35	0.18	0.35	0.44	0.32	0.36
Correlation with EMT marker: N-cadherin	0.47	0.20	0.31	0.44	0.29	0.08	0.48	0.05	0.09
Correlation with EMT marker: E-cadherin	0.09	0.04	-0.03	0.13	0.02	-0.11	0.13	0.07	0.20
Correlation with T_{reg} count	0.00	0.06	0.10	-0.01	-0.14	-0.23	-0.02	-0.15	-0.03
Correlation with neutrophil count	0.02	0.01	0.22*	-0.05	-0.17	0.05	-0.01	-0.07	-0.15
Correlation with MDSC count	-0.18	-0.32	-0.29	-0.13	0.04	-0.04	-0.15	-0.11	-0.15
Correlation with $CD8^+$ T cell count	0.08	-0.01	0.06	-0.13	0.04	0.26*	0.21	-0.03	0.04
Correlation with NK cell count	0.18	0.07	0.06	0.00	0.11	-0.05	0.10	-0.28*	-0.12
Correlation with conventional (myeloid) DC count	0.44	0.46	0.46	0.35	0.09	0.26	0.47	0.18	0.15
Correlation with plasmacytoid DC count	0.06	0.03	0.11	-0.16	-0.16	-0.07	-0.01	0.20	0.03
Correlation with endothelial cell count (marker of angiogenesis)	0.06	0.28	0.15	0.04	0.05	0.17	0.02	0.09	0.14*
Correlation with macrophage count	0.21	0.22	0.37	0.18	-0.14	0.14*	0.30	0.18	0.18*
Correlation with macrophage M1 count	0.31	0.36	0.40	0.15	-0.06	0.18	0.32	0.27*	0.19
Correlation with macrophage M2 count	0.21	0.25	0.23	0.18	-0.07	0.22	0.17	0.30	0.11

↑, red background - expression higher in tumor than in healthy tissue; ↓, blue background - expression lower in tumor than in healthy tissue; ↓, red background - worse prognosis; ↑, blue background – better prognosis; red background - positively correlated with the number of cells; blue background - negatively correlated with the number of cells.

Table S13. Involvement of CXCR2 ligands in kidney renal clear cell carcinoma (KIRC) tumorigenesis.

Name of the cancer	*CXCL1*	*CXCL2*	*CXCL3*	*CXCL5*	*CXCL6*	*PPBP*	*CXCL8*	*CXCR1*	*CXCR2*
Expression level relative to healthy tissue	=	=	=	=	=	=	=	=	=
Impact of elevated expression on prognosis	↓	↓	↓	↓	↓	=	↓	↑	↑
Link to lymph node metastasis status	=	=	=	=	=	↑	=	=	=
Correlation with proliferation	0.05	0.03	0.10	0.19	0.03	0.06	0.14	0.08	0.19
Correlation with EMT marker: vimentin	0.09	0.11	0.11	0.25	0.15	0.12	0.13	0.10	0.14
Correlation with EMT marker: N-cadherin	0.03	-0.01	0.00	0.08	0.08	0.12	0.14	0.31	0.42
Correlation with EMT marker: E-cadherin	-0.10	-0.13	-0.11	-0.08	-0.03	-0.10	-0.07	0.06	0.17
Correlation with T_{reg} count	-0.01	-0.05	-0.03	-0.01	0.05	-0.05	-0.02	-0.04	-0.04
Correlation with neutrophil count	0.10	0.14	0.17	0.00	0.04*	0.20	0.21	0.29	0.25
Correlation with MDSC count	0.08	0.09	0.05	0.09	0.05	0.04	0.00	-0.18	-0.32
Correlation with $CD8^+$ T cell count	-0.11	-0.07	-0.09	-0.06	-0.15	-0.06	-0.15	-0.10*	-0.05
Correlation with NK cell count	-0.01	0.00	0.05	0.01	-0.04	-0.02	-0.05*	0.01	-0.04
Correlation with conventional (myeloid) DC count	0.11*	0.08	0.17	0.21	0.13	0.03	0.36	0.13	0.24
Correlation with plasmacytoid DC count	0.05	-0.03	-0.06	0.04	0.03	-0.06	-0.02	-0.10	-0.06
Correlation with endothelial cell count (marker of angiogenesis)	-0.04	0.08	0.05	-0.05	0.06	0.25	-0.04	0.17	0.09*
Correlation with macrophage count	0.03	-0.09	0.02	0.14	0.02	-0.10	0.24	-0.02	0.13
Correlation with macrophage M1 count	0.09	-0.07	0.05	0.16	0.05	-0.07	0.24	-0.01	0.09
Correlation with macrophage M2 count	0.03	-0.07	-0.03	0.00	-0.01	-0.05	0.11	0.01	0.12

↑, red background - expression higher in tumor than in healthy tissue; ↓, blue background - expression lower in tumor than in healthy tissue; ↓, red background - worse prognosis; ↑, blue background – better prognosis; red background - positively correlated with the number of cells; blue background - negatively correlated with the number of cells.

Table S14. Involvement of CXCR2 ligands in kidney renal papillary cell carcinoma (KIRP) tumorigenesis.

Name of the cancer	*CXCL1*	*CXCL2*	*CXCL3*	*CXCL5*	*CXCL6*	*PPBP*	*CXCL8*	*CXCR1*	*CXCR2*
Expression level relative to healthy tissue	↑	=	=	=	↑	=	↑	=	=
Impact of elevated expression on prognosis	=	↑ p=0.072	=	↑ p=0.078	=	=	=	=	=
Link to lymph node metastasis status	=	=	=	=	↓	=	=	=	=
Correlation with proliferation	-0.06	-0.05	-0.02	0.00	-0.07	0.00	0.14	-0.03	0.19
Correlation with EMT marker: vimentin	0.32	0.20	0.31	0.29	0.35	0.25	0.42	0.25	0.30
Correlation with EMT marker: N-cadherin	0.15	0.05	0.09	0.27	0.32	0.19	0.22	0.11	0.24
Correlation with EMT marker: E-cadherin	-0.04	0.03	0.02	-0.05	0.00	0.04	-0.06	0.01	0.12
Correlation with T_{reg} count	-0.13*	-0.02	-0.11	-0.23*	-0.19*	-0.04	-0.07	-0.10*	-0.05*
Correlation with neutrophil count	0.04	-0.06	0.07	0.09*	0.12*	0.12	0.12	0.18	0.15
Correlation with MDSC count	-0.15	-0.24	-0.16	-0.11	-0.08	-0.07	-0.15	-0.19	-0.24
Correlation with $CD8^{+}$ T cell count	0.06	0.09	0.13	0.10	0.07	0.00	0.02*	0.05	0.08*
Correlation with NK cell count	-0.03*	0.07	0.00*	-0.06	-0.06	-0.16*	-0.11	-0.14*	-0.13*
Correlation with conventional (myeloid) DC count	0.32	0.31	0.33	0.32	0.27	0.29	0.35	0.29	0.34
Correlation with plasmacytoid DC count	0.04	0.11	0.11	0.03	-0.01	-0.07	0.02	0.05	0.02
Correlation with endothelial cell count (marker of angiogenesis)	-0.23	-0.11*	-0.16	-0.26	-0.34	0.00	0.00	0.09	0.03
Correlation with macrophage count	0.14	0.23	0.17	0.10*	0.09*	0.09*	0.20	0.16	0.21
Correlation with macrophage M1 count	0.17	0.25	0.21	0.07	0.10	0.10	0.22	0.15	0.20
Correlation with macrophage M2 count	0.09	0.17	0.03	0.01	0.04	0.02	0.07	0.08	0.08

↑, red background - expression higher in tumor than in healthy tissue; ↓, blue background - expression lower in tumor than in healthy tissue; ↓, red background - worse prognosis; ↑, blue background – better prognosis; red background - positively correlated with the number of cells; blue background - negatively correlated with the number of cells.

Table S15. Involvement of CXCR2 ligands in acute myeloid leukemia (LAML) tumorigenesis.

Name of the cancer	*CXCL1*	*CXCL2*	*CXCL3*	*CXCL5*	*CXCL6*	*PPBP*	*CXCL8*	*CXCR1*	*CXCR2*
Expression level relative to healthy tissue	=	=	↓	=	=	↑	=	=	↑
Impact of elevated expression on prognosis	↓	↓ p=0.055	=	↓	=	↓ P=0.099	=	=	=
Relation to lymph node metastasis status	N/A	N/A	N/A	N/A	N/A	N/A	N/A	N/A	N/A
Correlation with proliferation	0.04	-0.09	-0.05	-0.01	-0.02	0.08	0.00	0.11	0.29
Correlation with vimentin marker	0.02	-0.06	-0.11	-0.14	-0.07	-0.15	0.02	0.08	0.16
Correlation with N-cadherin marker	0.03	0.15	0.13	0.17	0.02	0.02	-0.05	-0.02	-0.25
Correlation with E-cadherin marker	0.23	0.13	0.17	0.36	0.07	0.39	0.00	0.21	-0.01
Correlation with T_{reg} count	N/A	N/A	N/A	N/A	N/A	N/A	N/A	N/A	N/A
Correlation with neutrophil count	N/A	N/A	N/A	N/A	N/A	N/A	N/A	N/A	N/A
Correlation with MDSC count	N/A	N/A	N/A	N/A	N/A	N/A	N/A	N/A	N/A
Correlation with $CD8^+$ T cell count	N/A	N/A	N/A	N/A	N/A	N/A	N/A	N/A	N/A
Correlation with NK cell count	N/A	N/A	N/A	N/A	N/A	N/A	N/A	N/A	N/A
Correlation with conventional (myeloid) DC count	N/A	N/A	N/A	N/A	N/A	N/A	N/A	N/A	N/A
Correlation with plasmacytoid DC count	N/A	N/A	N/A	N/A	N/A	N/A	N/A	N/A	N/A
Correlation with endothelial cell count (marker of angiogenesis)	N/A	N/A	N/A	N/A	N/A	N/A	N/A	N/A	N/A
Correlation with macrophage count	N/A	N/A	N/A	N/A	N/A	N/A	N/A	N/A	N/A
Correlation with macrophage M1 count	N/A	N/A	N/A	N/A	N/A	N/A	N/A	N/A	N/A
Correlation with macrophage M2 count	N/A	N/A	N/A	N/A	N/A	N/A	N/A	N/A	N/A

↑, red background - expression higher in tumor than in healthy tissue; ↓, blue background - expression lower in tumor than in healthy tissue; ↓, red background - worse prognosis; ↑, blue background – better prognosis; red background - positively correlated with the number of cells; blue background - negatively correlated with the number of cells.

Table S16. Involvement of CXCR2 ligands in brain lower grade glioma (LGG) tumorigenesis.

Name of the cancer	*CXCL1*	*CXCL2*	*CXCL3*	*CXCL5*	*CXCL6*	*PPBP*	*CXCL8*	*CXCR1*	*CXCR2*
Expression level relative to healthy tissue	=	=	=	=	=	=	=	=	=
Impact of elevated expression on prognosis	↓	↑	↑ p=0.096	↑	=	=	=	↓ p=0.075	↓
Link to lymph node metastasis status	N/A	N/A	N/A	N/A	N/A	N/A	N/A	N/A	N/A
Correlation with proliferation	-0.09	-0.13	-0.16	-0.24	-0.10	-0.07	-0.02	0.04	0.19
Correlation with EMT marker: vimentin	0.04	0.17	0.11	-0.09	0.18	0.02	0.16	0.11	0.46
Correlation with EMT marker: N-cadherin	-0.04	-0.04	-0.02	0.09	0.06	-0.05	0.11	0.08	0.27
Correlation with EMT marker: E-cadherin	-0.13	0.23	0.22	0.17	0.10	-0.04	-0.05	0.03	0.15
Correlation with T_{reg} count	0.05	-0.01*	-0.02	0.04*	-0.03	-0.08	0.07	0.11	-0.02
Correlation with neutrophil count	0.12	0.18*	0.20	0.14*	0.09	0.08	0.18	0.20	0.29
Correlation with MDSC count	0.01	-0.24	-0.28	-0.25	-0.15	0.00	-0.09	-0.05	-0.27
Correlation with $CD8^{+}$ T cell count	-0.04	0.05	0.10*	0.00	0.05	-0.04	-0.04	-0.03	0.01
Correlation with NK cell count	0.04	0.03*	0.11	0.10	-0.04	0.01	0.07	-0.02	-0.04
Correlation with conventional (myeloid) DC count	0.09	-0.05	-0.07	-0.06	0.02	-0.08	0.05	-0.06	-0.02*
Correlation with plasmacytoid DC count	0.00	-0.08	-0.02	-0.02	-0.05	0.06	-0.02	0.09	0.16
Correlation with endothelial cell count (marker of angiogenesis)	0.18	-0.14*	-0.17	-0.29	-0.09	0.20	0.13	0.19	0.13
Correlation with macrophage count	0.01	0.11	0.14	-0.08	0.14	0.02	0.17	0.08	0.50
Correlation with macrophage M1 count	0.02	0.09	0.12	-0.17*	0.12	0.02	0.17	0.07	0.48
Correlation with macrophage M2 count	0.07	-0.05	-0.18	0.01	-0.01	0.08	0.09	0.06	0.17

↑, red background - expression higher in tumor than in healthy tissue; ↓, blue background - expression lower in tumor than in healthy tissue; ↓, red background - worse prognosis; ↑, blue background – better prognosis; red background - positively correlated with the number of cells; blue background - negatively correlated with the number of cells.

Table S17. Involvement of CXCR2 ligands in liver hepatocellular carcinoma (LIHC) tumorigenesis.

Name of the cancer	*CXCL1*	*CXCL2*	*CXCL3*	*CXCL5*	*CXCL6*	*PPBP*	*CXCL8*	*CXCR1*	*CXCR2*
Expression level relative to healthy tissue	=	↓	=	=	=	=	=	=	=
Impact of elevated expression on prognosis	↓	=	↓	↓	↓	=	↓	=	=
Link to lymph node metastasis status	=	=	=	↑	=	N/A	=	=	=
Correlation with proliferation	0.20	-0.12	0.30	0.29	0.15	-0.11	0.20	0.00	0.09
Correlation with EMT marker: vimentin	0.43	0.18	0.39	0.34	0.46	0.17	0.44	0.29	0.42
Correlation with EMT marker: N-cadherin	0.15	0.10	0.20	0.08	0.14	0.00	0.20	0.15	0.28
Correlation with EMT marker: E-cadherin	0.20	0.11	0.15	0.13	0.21	-0.03	0.14	0.05	0.16
Correlation with T_{reg} count	-0.02*	0.10	-0.01*	-0.12*	-0.04*	-0.09	0.07*	0.05	0.06
Correlation with neutrophil count	0.11	0.12	0.12	0.18	0.12	0.07	0.13	0.18	0.21
Correlation with MDSC count	0.22	-0.19	0.28	0.33	0.23	-0.11	0.20	-0.04	-0.02
Correlation with $CD8^+$ T cell count	-0.15*	-0.05	-0.02*	-0.14*	-0.21*	-0.08	-0.18*	-0.04	-0.06
Correlation with NK cell count	0.03	0.03	0.05	0.06	0.08	-0.06	0.01	-0.11*	-0.09
Correlation with conventional (myeloid) DC count	0.13	0.04	0.14	0.06	0.03	-0.14*	0.07	0.03	0.06
Correlation with plasmacytoid DC count	-0.09	-0.05	-0.03	-0.14	-0.18	-0.05	-0.17	0.08	0.07
Correlation with endothelial cell count (marker of angiogenesis)	-0.24*	0.07	-0.28	-0.31	-0.21*	0.08	-0.25*	0.03	-0.03
Correlation with macrophage count	0.04	0.14	0.02	0.01	0.03	-0.02	0.05	0.04	0.08
Correlation with macrophage M1 count	0.23	0.02	0.18	0.18	0.16	-0.08	0.18	0.05	0.06
Correlation with macrophage M2 count	-0.10	0.16	-0.17	-0.19	-0.05	0.02	-0.12*	0.01	0.03

↑, red background - expression higher in tumor than in healthy tissue; ↓, blue background - expression lower in tumor than in healthy tissue; ↓, red background - worse prognosis; ↑, blue background – better prognosis; red background - positively correlated with the number of cells; blue background - negatively correlated with the number of cells.

Table S18. Involvement of CXCR2 ligands in lung adenocarcinoma (LUAD) tumorigenesis.

Name of the cancer	*CXCL1*	*CXCL2*	*CXCL3*	*CXCL5*	*CXCL6*	*PPBP*	*CXCL8*	*CXCR1*	*CXCR2*
Expression level relative to healthy tissue	=	↓	↓	=	=	↓	=	↓	↓
Impact of elevated expression on prognosis	=	=	=	=	↓ p=0.076	=	↓	=	=
Link to lymph node metastasis status	=	=	=	=	=	=	=	=	=
Correlation with proliferation	0.08	-0.09	0.08	0.16	0.10	-0.11	0.23	-0.07	-0.08
Correlation with EMT marker: vimentin	0.19	0.11	0.14	0.14	0.21	0.27	0.13	0.17	0.30
Correlation with EMT marker: N-cadherin	0.16	-0.03	0.10	0.24	0.27	0.04	0.27	0.16	0.13
Correlation with EMT marker: E-cadherin	0.00	-0.02	-0.06	-0.03	0.02	0.09	0.01	-0.07	0.02
Correlation with T_{reg} count	0.04	0.08	0.07	0.05	-0.06	-0.01	0.11*	0.07	0.13
Correlation with neutrophil count	0.13	0.18	0.19	0.19	0.10	0.23	0.21	0.40	0.33
Correlation with MDSC count	0.10	-0.12	0.05	0.13	0.12	-0.07	0.32	-0.16	-0.37
Correlation with $CD8^+$ T cell count	-0.12*	-0.01	-0.09	-0.17	-0.08	-0.23	-0.21	-0.10*	-0.11*
Correlation with NK cell count	-0.01	0.01	0.00	-0.06	-0.07	-0.05	-0.04	-0.08	-0.04
Correlation with conventional (myeloid) DC count	0.01	0.11	-0.01	0.01	0.03	0.25	-0.01	0.06	0.34
Correlation with plasmacytoid DC count	0.08	0.04	0.13	0.09	0.08	-0.17	0.15	0.03	-0.03
Correlation with endothelial cell count (marker of angiogenesis)	0.08	0.17	0.18	0.16	0.01	0.28	0.05	0.43	0.31
Correlation with macrophage count	-0.03	0.00	0.03	0.08	0.06*	0.08	0.11	0.09*	0.22
Correlation with macrophage M1 count	0.02	-0.06	0.04	0.08	0.12	-0.02	0.16	0.09	0.11
Correlation with macrophage M2 count	-0.15	0.06	-0.02	0.05	-0.10	0.19	-0.11	0.13	0.26

↑, red background - expression higher in tumor than in healthy tissue; ↓, blue background - expression lower in tumor than in healthy tissue; ↓, red background - worse prognosis; ↑, blue background – better prognosis; red background - positively correlated with the number of cells; blue background - negatively correlated with the number of cells.

Table S19. Involvement of CXCR2 ligands in lung squamous cell carcinoma (LUSC) tumorigenesis.

Name of the cancer	*CXCL1*	*CXCL2*	*CXCL3*	*CXCL5*	*CXCL6*	*PPBP*	*CXCL8*	*CXCR1*	*CXCR2*
Expression level relative to healthy tissue	=	↓	↓	↓	↑	↓	=	↓	↓
Impact of elevated expression on prognosis	=	↓	=	=	=	=	=	=	=
Link to lymph node metastasis status	=	↓	↓	=	=	??	=	??	↓
Correlation with proliferation	-0.08	-0.16	-0.10	-0.09	-0.13	-0.14	-0.16	-0.16	-0.10
Correlation with EMT marker: vimentin	0.06	0.29	0.27	0.39	0.07	0.33	0.06	0.24	0.09
Correlation with EMT marker: N-cadherin	-0.01	0.07	0.10	0.15	0.02	0.17	0.02	0.11	0.07
Correlation with EMT marker: E-cadherin	-0.09	-0.13	-0.20	-0.15	-0.07	-0.08	-0.15	-0.10	0.04
Correlation with T_{reg} count	0.02	0.14	0.13	0.10	0.02	-0.02	0.08	0.09	0.02
Correlation with neutrophil count	0.31	0.22	0.30	0.15	0.19	0.19	0.40	0.48	0.42
Correlation with MDSC count	0.20	-0.16	0.00	-0.05	0.15	-0.09	0.03	-0.19	-0.29
Correlation with $CD8^+$ T cell count	-0.26	-0.09	-0.11*	-0.07	-0.17	-0.11*	-0.15	-0.14*	-0.05
Correlation with NK cell count	-0.02	-0.06	-0.03	-0.12	-0.06	-0.11*	-0.02	-0.03	0.04
Correlation with conventional (myeloid) DC count	-0.04	0.16	0.13	0.25	0.04	0.18	0.15*	0.25	0.20
Correlation with plasmacytoid DC count	-0.16	-0.03	0.02	0.10	-0.18	-0.09	-0.02	-0.03	-0.08
Correlation with endothelial cell count (marker of angiogenesis)	-0.10	0.29	0.13	0.19	-0.05	0.26	-0.04	0.25	0.11
Correlation with macrophage count	-0.26	0.08*	0.01	0.16	-0.24	0.07	0.03	0.10	0.10*
Correlation with macrophage M1 count	-0.26	0.05	-0.01	0.15	-0.23*	0.02	-0.01	0.08	0.05
Correlation with macrophage M2 count	-0.27	0.13	-0.03	0.06	-0.24	0.12	-0.02	0.11	0.15

↑, red background - expression higher in tumor than in healthy tissue; ↓, blue background - expression lower in tumor than in healthy tissue; ↓, red background - worse prognosis; ↑, blue background – better prognosis; red background - positively correlated with the number of cells; blue background - negatively correlated with the number of cells.

Table S20. Involvement of CXCR2 ligands in ovarian serous cystadenocarcinoma (OV) tumorigenesis.

Name of the cancer	*CXCL1*	*CXCL2*	*CXCL3*	*CXCL5*	*CXCL6*	*PPBP*	*CXCL8*	*CXCR1*	*CXCR2*
Expression level relative to healthy tissue	↑	=	=	=	=	=	↑	=	=
Impact of elevated expression on prognosis	=	=	=	↑ p=0.083	=	=	=	=	=
Link to lymph node metastasis status	N/A	N/A	N/A	N/A	N/A	N/A	N/A	N/A	N/A
Correlation with proliferation	0.07	0.07	0.09	0.13	0.15	0.07	0.14	0.16	0.26
Correlation with EMT marker: vimentin	0.15	0.16	0.13	0.19	0.21	0.21	0.17	0.31	0.30
Correlation with EMT marker: N-cadherin	-0.06	0.00	-0.03	0.12	0.06	0.10	0.07	0.11	0.03
Correlation with EMT marker: E-cadherin	0.04	-0.05	-0.02	0.10	0.09	0.00	0.08	0.28	0.35
Correlation with T_{reg} count	-0.03	-0.02	-0.04	-0.11	-0.02	-0.05	-0.02	0.01	0.05
Correlation with neutrophil count	0.08	0.02	0.10	-0.01	0.09	0.06	0.09	0.12*	0.10
Correlation with MDSC count	-0.06	-0.06	-0.05	0.05	-0.03	0.11	0.00	-0.23	-0.42
Correlation with $CD8^+$ T cell count	-0.10	-0.15	-0.11	-0.08	-0.03	-0.26	-0.13*	-0.10	0.00
Correlation with NK cell count	0.01	-0.06	-0.04	-0.13	-0.03	-0.14	-0.03	-0.21*	-0.09
Correlation with conventional (myeloid) DC count	0.22	0.17	0.17	0.11	0.20	-0.04	0.21	0.16	0.14
Correlation with plasmacytoid DC count	0.17	0.01	0.06	0.02	0.00	-0.12	0.10	-0.08	-0.07
Correlation with endothelial cell count (marker of angiogenesis)	-0.04	0.06	0.00	-0.05	-0.04	0.16	-0.06	0.12*	0.07*
Correlation with macrophage count	0.08	0.04	0.06	0.05	0.06	-0.14	0.15*	0.13	0.30
Correlation with macrophage M1 count	0.14	0.08	0.11	0.08	0.11	-0.10	0.14	0.13	0.31
Correlation with macrophage M2 count	-0.14	-0.09	-0.11	-0.13*	-0.12	-0.18	-0.03	0.02	0.23

↑, red background - expression higher in tumor than in healthy tissue; ↓, blue background - expression lower in tumor than in healthy tissue; ↓, red background - worse prognosis; ↑, blue background – better prognosis; red background - positively correlated with the number of cells; blue background - negatively correlated with the number of cells.

Table S21. Involvement of CXCR2 ligands in pancreatic adenocarcinoma (PAAD) tumorigenesis.

Name of the cancer	*CXCL1*	*CXCL2*	*CXCL3*	*CXCL5*	*CXCL6*	*PPBP*	*CXCL8*	*CXCR1*	*CXCR2*
Expression level relative to healthy tissue	↑	=	↑	↑	↑	=	↑	=	=
Impact of elevated expression on prognosis	=	=	=	↓	=	=	=	=	=
Link to lymph node metastasis status	=	=	=	=	=	=	=	=	=
Correlation with proliferation	-0.02	-0.09	0.13	0.18	-0.08	0.01	0.10	0.02	-0.04
Correlation with EMT marker: vimentin	0.21	0.24	0.03	0.16	0.19	0.38	0.34	0.31	0.42
Correlation with EMT marker: N-cadherin	0.13	0.04	-0.07	0.13	0.16	0.25	0.25	0.15	0.24
Correlation with EMT marker: E-cadherin	0.06	-0.09	0.18	0.14	0.12	0.06	0.21	0.01	0.01
Correlation with T_{reg} count	-0.14*	-0.05*	0.01	0.10	-0.14	0.14*	0.02*	0.13	0.17
Correlation with neutrophil count	0.17	0.18	0.15	0.28	-0.01*	0.05*	0.23	0.26	0.25
Correlation with MDSC count	-0.05	-0.17	-0.05	0.00	-0.14	-0.04	-0.02	-0.24	-0.44
Correlation with $CD8^{+}$ T cell count	0.09	0.12	0.00	-0.05	0.16	-0.01	0.04	0.21	0.31
Correlation with NK cell count	-0.15	-0.22*	-0.22*	-0.07	0.00	-0.13	-0.23*	-0.17*	-0.08
Correlation with conventional (myeloid) DC count	0.18	0.23	0.17	0.14	0.12	0.33	0.27	0.32	0.47
Correlation with plasmacytoid DC count	-0.07	-0.07	0.04	0.18	-0.05	-0.09	-0.03	0.13	0.09
Correlation with endothelial cell count (marker of angiogenesis)	0.12*	0.21	-0.06	0.03	0.06*	0.12*	0.05	0.26	0.35
Correlation with macrophage count	0.00	0.05	-0.03	0.19	0.13	0.04	0.14*	0.19	0.33
Correlation with macrophage M1 count	-0.04	-0.01	-0.09	0.13*	0.08	-0.04	0.05	0.09	0.20
Correlation with macrophage M2 count	0.00	0.04	-0.03	0.16	0.04	0.04	0.05	0.27	0.39

↑, red background - expression higher in tumor than in healthy tissue; ↓, blue background - expression lower in tumor than in healthy tissue; ↓, red background - worse prognosis; ↑, blue background – better prognosis; red background - positively correlated with the number of cells; blue background - negatively correlated with the number of cells.

Table S22. Involvement of CXCR2 ligands in pheochromocytoma and paraganglioma (PCPG) tumorigenesis.

Name of the cancer	*CXCL1*	*CXCL2*	*CXCL3*	*CXCL5*	*CXCL6*	*PPBP*	*CXCL8*	*CXCR1*	*CXCR2*
Expression level relative to healthy tissue	↓	=	=	=	=	=	↓	=	=
Impact of elevated expression on prognosis	=	=	=	=	N/A	=	=	=	=
Link to lymph node metastasis status	N/A	N/A	N/A	N/A	N/A	N/A	N/A	N/A	N/A
Correlation with proliferation	-0.03	-0.10	0.03	-0.07	0.09	0.02	0.05	0.14	0.16
Correlation with EMT marker: vimentin	0.09	0.17	0.20	0.04	0.04	0.14	0.17	0.08	0.06
Correlation with EMT marker: N-cadherin	0.20	0.13	0.23	0.28	0.16	0.07	0.24	0.16	0.30
Correlation with EMT marker: E-cadherin	0.19	0.13	0.22	0.16	0.11	0.12	0.09	0.13	0.11
Correlation with T_{reg} count	0.13	0.08	0.15	0.15*	0.04	0.05	0.20	0.11	0.04
Correlation with neutrophil count	0.18	0.20	0.17	0.22	0.18	0.30	0.21	0.36	0.36
Correlation with MDSC count	-0.49	-0.43	-0.38	-0.33	-0.13	-0.36	-0.40	-0.27	-0.32
Correlation with $CD8^+$ T cell count	-0.06	-0.14	-0.06	-0.12	-0.06	-0.09	-0.15*	-0.09	-0.08
Correlation with NK cell count	0.01	0.00	0.04	-0.03	-0.11	-0.01	0.04	-0.02	-0.05
Correlation with conventional (myeloid) DC count	0.41	0.39	0.38	0.21	0.08	0.23	0.39	0.16	0.21
Correlation with plasmacytoid DC count	-0.06	-0.07	-0.09	-0.06	-0.07	0.02	-0.07	-0.04	0.02
Correlation with endothelial cell count (marker of angiogenesis)	0.02	0.05	0.01	-0.13	0.01	0.17	0.03	0.01	0.00
Correlation with macrophage count	0.39	0.35	0.34	0.32	0.16	0.25	0.39	0.21	0.29
Correlation with macrophage M1 count	0.46	0.41	0.39	0.34	0.21	0.29	0.41	0.27	0.32
Correlation with macrophage M2 count	0.12	0.21	0.19	0.01	0.00	0.14	0.19	0.11	0.17

↑, red background - expression higher in tumor than in healthy tissue; ↓, blue background - expression lower in tumor than in healthy tissue; ↓, red background - worse prognosis; ↑, blue background – better prognosis; red background - positively correlated with the number of cells; blue background - negatively correlated with the number of cells.

Table S23. Involvement of CXCR2 ligands in prostate adenocarcinoma (PRAD) tumorigenesis.

Name of the cancer	*CXCL1*	*CXCL2*	*CXCL3*	*CXCL5*	*CXCL6*	*PPBP*	*CXCL8*	*CXCR1*	*CXCR2*
Expression level relative to healthy tissue	=	↓	=	=	=	=	=	=	=
Impact of elevated expression on prognosis	=	=	=	=	=	N/A	=	=	=
Link to lymph node metastasis status	=	↓	↓ p=0.059	↓	=	=	=	↓	↓
Correlation with proliferation	-0.01	-0.01	0.05	0.05	0.00	0.14	0.21	0.08	0.03
Correlation with EMT marker: vimentin	0.26	0.29	0.25	0.24	0.33	0.22	0.27	0.32	0.36
Correlation with EMT marker: N-cadherin	0.13	0.16	0.17	0.14	0.21	0.14	0.18	0.23	0.29
Correlation with EMT marker: E-cadherin	0.08	0.04	0.06	0.19	0.16	0.05	0.23	0.23	0.27
Correlation with T_{reg} count	-0.05	-0.02	-0.02	-0.08	-0.03	-0.04	0.10	0.08	0.01
Correlation with neutrophil count	0.06*	0.07*	0.08*	0.03*	0.07	0.08	0.08*	0.12	0.11
Correlation with MDSC count	-0.24	-0.23	-0.21	-0.22	-0.21	-0.06	-0.14	-0.32	-0.40
Correlation with $CD8^+$ T cell count	0.02	-0.04	0.01	0.04	0.00*	-0.04	-0.05*	-0.07	-0.04*
Correlation with NK cell count	-0.01	0.02	0.00	-0.01	-0.01	-0.02	0.03	-0.03	-0.05
Correlation with conventional (myeloid) DC count	0.20	0.16	0.18	0.16	0.13	0.08*	0.16	0.16	0.12
Correlation with plasmacytoid DC count	0.05	0.05	0.06	0.04	-0.04	0.07	0.08	-0.02	-0.04
Correlation with endothelial cell count (marker of angiogenesis)	0.01*	0.06*	-0.04	-0.05	0.02*	0.14	-0.01*	0.16	0.02*
Correlation with macrophage count	0.06	0.07	0.09	0.09	0.04*	0.05	0.08	0.10	0.00*
Correlation with macrophage M1 count	0.10	0.11	0.12	0.03	0.06	0.11	0.00	0.10	0.05
Correlation with macrophage M2 count	-0.06	-0.01	-0.05	-0.08	-0.10*	0.04	-0.05	0.00	-0.12

↑, red background - expression higher in tumor than in healthy tissue; ↓, blue background - expression lower in tumor than in healthy tissue; ↓, red background - worse prognosis; ↑, blue background – better prognosis; red background - positively correlated with the number of cells; blue background - negatively correlated with the number of cells.

Table S24. Involvement of CXCR2 ligands in rectum adenocarcinoma (READ) tumorigenesis.

Name of the cancer	*CXCL1*	*CXCL2*	*CXCL3*	*CXCL5*	*CXCL6*	*PPBP*	*CXCL8*	*CXCR1*	*CXCR2*
Expression level relative to healthy tissue	↑	↑	↑	↑	=	↑	↑	=	=
Impact of elevated expression on prognosis	=	=	=	=	=	=	=	=	=
Link to lymph node metastasis status	↓	↓	↓	=	=	=	=	=	=
Correlation with proliferation	0.31	0.23	0.33	0.28	0.25	0.20	0.22	0.27	0.28
Correlation with EMT marker: vimentin	0.04	-0.04	-0.13	0.33	0.20	0.26	0.40	0.47	0.34
Correlation with EMT marker: N-cadherin	-0.23	-0.20	-0.35	0.13	0.07	0.21	0.20	0.23	0.10
Correlation with EMT marker: E-cadherin	0.03	-0.07	0.11	-0.07	0.02	0.04	-0.07	0.02	0.07
Correlation with T_{reg} count	0.14	0.24	0.21*	0.18	0.16	0.16	0.15	0.11*	0.20
Correlation with neutrophil count	0.16*	0.16	0.19	0.21	0.26	0.03	0.19*	0.40	0.36
Correlation with MDSC count	0.07	0.10	0.09	0.22	0.17	0.14	0.02	-0.24	-0.20
Correlation with $CD8^+$ T cell count	-0.01	0.14	-0.02	-0.02	-0.04	0.02	0.07	0.17	0.13
Correlation with NK cell count	0.11	0.05	0.04	-0.02	0.05	0.16	0.09	-0.01	0.07
Correlation with conventional (myeloid) DC count	0.08	0.03	-0.01	0.16	0.18	0.02	0.30	0.37	0.34
Correlation with plasmacytoid DC count	0.08	0.22	0.10	0.07	0.12	-0.07	0.09	0.01	0.06
Correlation with endothelial cell count (marker of angiogenesis)	0.24*	0.18	0.09	0.39	0.35	0.06	0.38	0.42	0.36
Correlation with macrophage count	-0.02	-0.11	-0.13	0.04	0.02	0.15	0.32	0.25	0.19
Correlation with macrophage M1 count	-0.02	-0.04	-0.12	0.17	0.12	0.09	0.31	0.31	0.27
Correlation with macrophage M2 count	-0.09	-0.23	-0.18	-0.11	-0.16	0.07	0.14	0.08	0.00

↑, red background - expression higher in tumor than in healthy tissue; ↓, blue background - expression lower in tumor than in healthy tissue; ↓, red background - worse prognosis; ↑, blue background – better prognosis; red background - positively correlated with the number of cells; blue background - negatively correlated with the number of cells.

Table S25. Involvement of CXCR2 ligands in sarcoma (SARC) tumorigenesis.

Name of the cancer	*CXCL1*	*CXCL2*	*CXCL3*	*CXCL5*	*CXCL6*	*PPBP*	*CXCL8*	*CXCR1*	*CXCR2*
Expression level relative to healthy tissue	=	=	=	=	=	=	=	=	=
Impact of elevated expression on prognosis	=	↑	=	=	=	↓ p=0.091	=	=	=
Link to lymph node metastasis status	N/A	N/A	N/A	N/A	N/A	N/A	N/A	N/A	N/A
Correlation with proliferation	0.08	0.00	0.16	0.19	0.05	0.02	0.21	-0.13	0.05
Correlation with EMT marker: vimentin	0.18	0.05	0.25	0.17	0.14	0.15	0.26	0.08	0.05
Correlation with EMT marker: N-cadherin	-0.13	-0.23	0.03	0.10	-0.05	0.04	0.07	-0.20	0.04
Correlation with EMT marker: E-cadherin	-0.32	-0.02	-0.18	-0.10	-0.19	-0.18	-0.23	-0.21	-0.09
Correlation with T_{reg} count	0.08	0.07*	0.11	-0.02	0.06	0.11	0.13	0.10	0.05
Correlation with neutrophil count	0.16	0.22	0.04*	0.02	0.09	0.16	0.17	0.13	0.14
Correlation with MDSC count	-0.20	-0.33	-0.08	0.06	-0.13	0.03	-0.02	-0.29	-0.32
Correlation with $CD8^+$ T cell count	0.05	0.11	0.10	0.00	0.02	-0.06	-0.03	-0.07	0.00
Correlation with NK cell count	-0.02	-0.07	-0.06	0.03	0.01	0.10	0.08	-0.02	0.03
Correlation with conventional (myeloid) DC count	0.46	0.46	0.36	0.27	0.34	0.12	0.25	0.27	0.30
Correlation with plasmacytoid DC count	0.11	0.23	0.26	0.15	0.10	0.02	0.02	0.07	0.06
Correlation with endothelial cell count (marker of angiogenesis)	0.26	0.26	0.04	-0.05	0.13	0.24	0.15	0.38	0.31
Correlation with macrophage count	0.40	0.21	0.39	0.30	0.30	0.12	0.32	0.23	0.24
Correlation with macrophage M1 count	0.41	0.28	0.41	0.30	0.32	0.11	0.32	0.22	0.29
Correlation with macrophage M2 count	0.15	0.19	0.09	0.04	0.04	-0.02	0.09*	0.23	0.15

↑, red background - expression higher in tumor than in healthy tissue; ↓, blue background - expression lower in tumor than in healthy tissue; ↓, red background - worse prognosis; ↑, blue background – better prognosis; red background - positively correlated with the number of cells; blue background - negatively correlated with the number of cells.

Table S26. Involvement of CXCR2 ligands in skin cutaneous melanoma (SKCM) tumorigenesis.

Name of the cancer	*CXCL1*	*CXCL2*	*CXCL3*	*CXCL5*	*CXCL6*	*PPBP*	*CXCL8*	*CXCR1*	*CXCR2*
Expression level relative to healthy tissue	↑	=	=	=	=	=	↑	=	↓
Impact of elevated expression on prognosis	=	=	=	=	=	↓ p=0.064	=	=	↓
Link to lymph node metastasis status	=	↑	=	=	=	=	=	=	↑
Correlation with proliferation	0.11	0.15	0.15	0.14	0.11	0.02	0.21	0.05	0.11
Correlation with EMT marker: vimentin	0.10	-0.05	0.01	0.03	0.02	0.01	0.10	-0.04	-0.02
Correlation with EMT marker: N-cadherin	-0.03	0.11	0.12	0.11	0.10	0.05	0.15	-0.08	0.04
Correlation with EMT marker: E-cadherin	0.22	-0.11	-0.10	-0.06	0.02	-0.07	-0.07	0.06	0.06
Correlation with T_{reg} count	0.16*	0.05	0.09	-0.04	-0.08	-0.02	0.22	-0.10*	-0.08
Correlation with neutrophil count	0.09*	0.05*	0.09	0.09	0.06	0.02	0.13	0.06	0.09*
Correlation with MDSC count	0.20	0.13	0.25	0.23	0.23	0.20	0.29	0.08	-0.01
Correlation with $CD8^{+}$ T cell count	-0.16	0.00	-0.11	-0.12	-0.19	-0.20	-0.14	-0.16	-0.10
Correlation with NK cell count	0.05	-0.02	-0.01	-0.08	-0.09	-0.08	0.02	-0.08	-0.08
Correlation with conventional (myeloid) DC count	-0.03	0.13	0.06	0.10	-0.04	-0.03	-0.04	0.06	0.12*
Correlation with plasmacytoid DC count	-0.03	0.04	-0.03	-0.05	-0.13	-0.22	-0.11	-0.10	-0.12
Correlation with endothelial cell count (marker of angiogenesis)	-0.23	0.04	-0.05	0.10*	0.05	0.17	-0.07	0.10	0.10
Correlation with macrophage count	-0.05*	0.01	-0.02	-0.08	-0.13*	-0.18	0.09	-0.12*	-0.02
Correlation with macrophage M1 count	-0.08	0.02	-0.03	-0.05	-0.11	-0.15	0.07	-0.09	0.02
Correlation with macrophage M2 count	-0.13	-0.17	-0.17	-0.25	-0.19*	-0.16	-0.19	-0.12*	-0.09

↑, red background - expression higher in tumor than in healthy tissue; ↓, blue background - expression lower in tumor than in healthy tissue; ↓, red background - worse prognosis; ↑, blue background – better prognosis; red background - positively correlated with the number of cells; blue background - negatively correlated with the number of cells.

Table S27. Involvement of CXCR2 ligands in tumorigenesis of stomal adenocarcinoma (STAD).

Name of the cancer	*CXCL1*	*CXCL2*	*CXCL3*	*CXCL5*	*CXCL6*	*PPBP*	*CXCL8*	*CXCR1*	*CXCR2*
Expression level relative to healthy tissue	↑	=	↑	↑	↑	=	↑	=	=
Impact of elevated expression on prognosis	=	=	↑ p=0.055	=	=	=	=	=	=
Link to lymph node metastasis status	↓	↓	↓	=	=	=	=	=	=
Correlation with proliferation	0.26	0.21	0.29	0.18	0.07	0.16	0.26	0.08	0.02
Correlation with EMT marker: vimentin	-0.05	-0.07	-0.17	0.01	0.08	-0.02	0.02	0.16	0.16
Correlation with EMT marker: N-cadherin	-0.13	-0.08	-0.27	-0.04	0.00	0.07	0.02	0.05	0.03
Correlation with EMT marker: E-cadherin	0.21	0.09	0.19	0.08	0.15	0.09	0.20	0.15	0.14
Correlation with T_{reg} count	0.13*	0.05	0.13*	0.06	0.02	-0.01	0.15*	0.11	0.18
Correlation with neutrophil count	0.27	0.26	0.25	0.32	0.27	0.26	0.40	0.56	0.53
Correlation with MDSC count	0.18	0.22	0.16	0.07	0.14	0.26	0.16	-0.17	-0.23
Correlation with $CD8^{+}$ T cell count	-0.18	-0.19	-0.12	-0.16	-0.24	-0.24	-0.22	-0.11	-0.10*
Correlation with NK cell count	0.02	0.04	0.05	0.02	-0.03	0.03	0.06	0.05	0.05
Correlation with conventional (myeloid) DC count	0.02	-0.09*	-0.02	-0.03	-0.04*	-0.06*	0.09	0.23	0.23
Correlation with plasmacytoid DC count	0.22	0.10	0.26	0.07	-0.07	0.03	0.20	0.15	0.11
Correlation with endothelial cell count (marker of angiogenesis)	-0.02	-0.01	-0.08*	0.14	0.16	0.01	0.04	0.16	0.15
Correlation with macrophage count	0.10	-0.01	0.11*	0.06	-0.08	-0.06	0.19	0.27	0.25
Correlation with macrophage M1 count	0.09	-0.01	0.08	0.05	-0.05	-0.01	0.17	0.27	0.23
Correlation with macrophage M2 count	0.02*	-0.03*	0.08*	0.09	-0.09*	-0.14	0.07	0.26	0.24

↑, red background - expression higher in tumor than in healthy tissue; ↓, blue background - expression lower in tumor than in healthy tissue; ↓, red background - worse prognosis; ↑, blue background – better prognosis; red background - positively correlated with the number of cells; blue background - negatively correlated with the number of cells.

Table S28. Involvement of CXCR2 ligands in testicular germ cell (TGCT) tumors.

Name of the cancer	*CXCL1*	*CXCL2*	*CXCL3*	*CXCL5*	*CXCL6*	*PPBP*	*CXCL8*	*CXCR1*	*CXCR2*
Expression level relative to healthy tissue	=	=	=	=	=	=	=	=	=
Impact of elevated expression on prognosis	=	=	=	=	=	N/A	=	=	=
Link to lymph node metastasis status	N/A	N/A	N/A	N/A	N/A	N/A	N/A	N/A	N/A
Correlation with proliferation	0.07	-0.06	0.11	0.05	0.08	-0.03	-0.06	-0.15	0.00
Correlation with EMT marker: vimentin	0.54	0.57	0.58	0.41	0.45	0.23	0.48	0.21	0.31
Correlation with EMT marker: N-cadherin	0.07	0.01	0.13	0.05	0.26	-0.04	-0.14	-0.14	-0.02
Correlation with EMT marker: E-cadherin	0.20	0.21	0.16	0.49	0.05	0.44	0.37	0.40	0.24
Correlation with T_{reg} count	-0.25*	-0.15*	-0.06*	-0.39*	-0.13*	-0.34*	-0.20*	-0.26*	-0.18*
Correlation with neutrophil count	0.02	0.05	0.11	0.25*	-0.05	0.04	0.24	0.09	0.17
Correlation with MDSC count	-0.15	-0.22	-0.16	0.15	-0.24	0.19	-0.05	0.10	-0.02
Correlation with $CD8^+$ T cell count	-0.17	-0.09	-0.14	-0.35*	-0.08	-0.31	-0.31	-0.20*	-0.18*
Correlation with NK cell count	-0.22	-0.22*	-0.22*	-0.27	-0.18*	-0.13	-0.21*	-0.09	-0.17*
Correlation with conventional (myeloid) DC count	0.37	0.42	0.35	0.20	0.33	0.01	0.35	0.20	0.30
Correlation with plasmacytoid DC count	-0.15	-0.11	-0.14	-0.25	-0.16	-0.30	-0.20	-0.18	-0.04
Correlation with endothelial cell count (marker of angiogenesis)	0.45	0.47	0.43	0.54	0.30	0.47	0.42	0.50	0.42
Correlation with macrophage count	0.27*	0.41	0.31	0.16	0.15	-0.03	0.48	0.13	0.23*
Correlation with macrophage M1 count	0.43	0.55	0.44	0.35	0.28	0.11	0.59	0.29	0.40
Correlation with macrophage M2 count	0.03	0.23	0.22	0.02*	-0.04	-0.06	0.24	0.01*	0.02*

↑, red background - expression higher in tumor than in healthy tissue; ↓, blue background - expression lower in tumor than in healthy tissue; ↓, red background - worse prognosis; ↑, blue background – better prognosis; red background - positively correlated with the number of cells; blue background - negatively correlated with the number of cells.

Table S29. Involvement of CXCR2 ligands in thyroid carcinoma (THCA) tumorigenesis.

Name of the cancer	*CXCL1*	*CXCL2*	*CXCL3*	*CXCL5*	*CXCL6*	*PPBP*	*CXCL8*	*CXCR1*	*CXCR2*
Expression level relative to healthy tissue	=	=	=	=	=	=	=	=	=
Impact of elevated expression on prognosis	=	=	=	=	=	↓	=	=	=
Link to lymph node metastasis status	↑ p=0.094	↑	↑ p=0.082	=	=	=	↑	=	=
Correlation with proliferation	0.41	0.34	0.37	0.43	0.28	0.24	0.39	0.12	0.41
Correlation with EMT marker: vimentin	0.02	0.21	0.16	0.04	0.09	0.11	0.13	0.22	0.13
Correlation with EMT marker: N-cadherin	-0.21	-0.09	-0.16	-0.28	-0.13	-0.06	-0.14	0.10	-0.07
Correlation with EMT marker: E-cadherin	0.05	0.10	0.11	0.12	0.06	0.12	0.15	0.10	0.11
Correlation with T_{reg} count	0.27	0.21	0.27	0.40	0.11	0.23	0.36	0.00	0.26
Correlation with neutrophil count	-0.08*	-0.02*	-0.03*	-0.05*	-0.05	0.06*	0.00	0.07	-0.03*
Correlation with MDSC count	-0.21	-0.12	-0.14	-0.18	-0.22	-0.02	-0.16	-0.17	-0.44
Correlation with $CD8^{+}$ T cell count	-0.01	-0.02	-0.03	-0.11*	0.11	-0.21	-0.08	0.10	0.18*
Correlation with NK cell count	0.06	0.01	0.04	-0.01	0.05*	0.00	0.04	0.02	-0.01
Correlation with conventional (myeloid) DC count	0.69	0.59	0.57	0.73	0.47	0.45	0.61	0.20	0.63
Correlation with plasmacytoid DC count	0.05	-0.03	0.00	-0.06	0.11	-0.12	-0.04	0.08	0.09
Correlation with endothelial cell count (marker of angiogenesis)	-0.38	-0.23	-0.31	-0.53	-0.18	-0.20	-0.40	0.10	-0.23
Correlation with macrophage count	0.26	0.17	0.22	0.29	0.17	0.07	0.25	0.01	0.29
Correlation with macrophage M1 count	0.17	0.16	0.17	0.11	0.14	-0.03	0.12	0.11	0.27
Correlation with macrophage M2 count	-0.21	-0.21	-0.19	-0.17	-0.06	-0.18	-0.16	-0.07	-0.05

↑, red background - expression higher in tumor than in healthy tissue; ↓, blue background - expression lower in tumor than in healthy tissue; ↓, red background - worse prognosis; ↑, blue background – better prognosis; red background - positively correlated with the number of cells; blue background - negatively correlated with the number of cells.

Table S30. Involvement of CXCR2 ligands in thymoma tumorigenesis (THYM).

Name of the cancer	*CXCL1*	*CXCL2*	*CXCL3*	*CXCL5*	*CXCL6*	*PPBP*	*CXCL8*	*CXCR1*	*CXCR2*
Expression level relative to healthy tissue	↓	=	=	=	=	↓	↓	↓	↓
Impact of elevated expression on prognosis	=	=	=	=	=	=	=	=	=
Link to lymph node metastasis status	N/A	N/A	N/A	N/A	N/A	N/A	N/A	N/A	N/A
Correlation with proliferation	-0.25	0.28	0.71	-0.07	-0.02	-0.25	0.32	-0.23	0.11
Correlation with EMT marker: vimentin	0.46	0.17	0.31	0.52	0.56	-0.12	0.32	0.19	0.30
Correlation with EMT marker: N-cadherin	0.46	0.03	0.04	0.48	0.53	0.00	-0.02	0.03	0.10
Correlation with EMT marker: E-cadherin	0.38	-0.01	-0.37	0.33	0.28	0.18	0.11	0.22	0.32
Correlation with T_{reg} count	0.20	0.16	0.16	0.17*	0.19	0.05	0.20*	-0.02	0.22
Correlation with neutrophil count	0.35	-0.09	-0.02	0.29	0.36*	0.05	0.15*	0.11	0.25
Correlation with MDSC count	0.21	-0.08	0.12	0.03	0.14	-0.24	0.12	-0.25	-0.03
Correlation with $CD8^{+}$ T cell count	-0.34	0.09	0.46	-0.12	-0.07	-0.09	0.09	-0.08	-0.10
Correlation with NK cell count	0.15	-0.06	0.02*	0.14	0.18	-0.01	0.12	0.18	-0.17
Correlation with conventional (myeloid) DC count	-0.54	0.08	0.23	-0.30*	-0.31*	-0.16	-0.10	-0.13	-0.23*
Correlation with plasmacytoid DC count	-0.55	0.05	0.11	-0.42	-0.46	0.02	-0.28	0.00	-0.18
Correlation with endothelial cell count (marker of angiogenesis)	0.33	0.00	-0.32	0.17*	0.20	0.28	-0.17	0.30	-0.15
Correlation with macrophage count	0.08	-0.04	-0.27	0.10	-0.06	0.26	-0.09	0.14	0.03
Correlation with macrophage M1 count	0.10	-0.02	-0.27	0.09	-0.03	0.19	-0.07	0.14	0.16
Correlation with macrophage M2 count	0.21	0.01	-0.31	0.15	0.06	0.22	-0.02	0.05*	0.01*

↑, red background - expression higher in tumor than in healthy tissue; ↓, blue background - expression lower in tumor than in healthy tissue; ↓, red background - worse prognosis; ↑, blue background – better prognosis; red background - positively correlated with the number of cells; blue background - negatively correlated with the number of cells.

Table S31. Involvement of CXCR2 ligands in uterine corpus endometrial carcinoma (UCEC) tumorigenesis.

Name of the cancer	*CXCL1*	*CXCL2*	*CXCL3*	*CXCL5*	*CXCL6*	*PPBP*	*CXCL8*	*CXCR1*	*CXCR2*
Expression level relative to healthy tissue	↑	=	↑	=	=	=	↑	=	=
Impact of elevated expression on prognosis	=	=	=	=	=	=	=	=	=
Link to lymph node metastasis status	N/A	N/A	N/A	N/A	N/A	N/A	N/A	N/A	N/A
Correlation with proliferation	0.00	-0.06	-0.02	0.08	0.08	-0.03	0.11	-0.12	0.05
Correlation with EMT marker: vimentin	0.17	0.17	0.18	0.18	0.11	0.01	0.17	0.16	0.22
Correlation with EMT marker: N-cadherin	-0.04	-0.07	0.01	0.12	0.15	0.03	0.03	-0.03	0.01
Correlation with EMT marker: E-cadherin	0.20	0.17	0.19	0.29	0.21	0.15	0.38	0.25	0.38
Correlation with T_{reg} count	0.08	0.17	0.17	-0.02	0.09	-0.03	0.05	0.03	-0.06*
Correlation with neutrophil count	0.15	0.19*	0.14	0.21*	0.19	0.11	0.30	0.37	0.18*
Correlation with MDSC count	-0.02	-0.23	-0.12	-0.14	0.00	-0.02	0.01	-0.35	-0.40
Correlation with $CD8^+$ T cell count	-0.07	-0.01	-0.05	-0.15	-0.07	-0.08	-0.26*	0.03*	0.02
Correlation with NK cell count	-0.25*	0.09	-0.02	-0.04	-0.25*	0.11	-0.15	0.00	-0.09
Correlation with conventional (myeloid) DC count	0.09	0.14	0.04	-0.02	-0.10	0.01	0.00	0.40	0.26
Correlation with plasmacytoid DC count	-0.05	0.02	-0.07	-0.10	-0.15	-0.07	-0.25	0.18	0.04
Correlation with endothelial cell count (marker of angiogenesis)	-0.27*	-0.21*	-0.26	-0.20	0.04	0.04	-0.18	-0.11	-0.18
Correlation with macrophage count	-0.04	0.01	-0.13	-0.28*	-0.14	-0.17	-0.05	0.13	-0.03
Correlation with macrophage M1 count	-0.05	-0.06	-0.17	-0.32*	-0.10	-0.16	-0.14	0.04	-0.13
Correlation with macrophage M2 count	-0.10	-0.09	-0.21	-0.31	-0.08	-0.13	-0.15	0.03	-0.06

↑, red background - expression higher in tumor than in healthy tissue; ↓, blue background - expression lower in tumor than in healthy tissue; ↓, red background - worse prognosis; ↑, blue background – better prognosis; red background - positively correlated with the number of cells; blue background - negatively correlated with the number of cells.

Table S32. Involvement of CXCR2 ligands in uterine carcinosarcoma (UCS) tumorigenesis.

Name of the cancer	*CXCL1*	*CXCL2*	*CXCL3*	*CXCL5*	*CXCL6*	*PPBP*	*CXCL8*	*CXCR1*	*CXCR2*
Expression level relative to healthy tissue	↑	=	=	=	=	=	↑	=	=
Impact of elevated expression on prognosis	=	=	=	=	=	=	=	=	=
Link to lymph node metastasis status	N/A	N/A	N/A	N/A	N/A	N/A	N/A	N/A	N/A
Correlation with proliferation	-0.02	0.06	0.08	0.33	-0.04	0.04	0.18	-0.03	0.09
Correlation with EMT marker: vimentin	0.17	0.30	0.26	0.35	0.07	0.19	0.32	0.26	0.11
Correlation with EMT marker: N-cadherin	0.24	0.28	0.24	0.23	-0.08	0.31	0.22	0.13	0.19
Correlation with EMT marker: E-cadherin	0.55	0.20	0.30	0.19	0.13	0.09	0.32	0.22	0.18
Correlation with T_{reg} count	-0.08*	-0.05	-0.07*	-0.18	0.12	-0.29*	-0.20	0.07	-0.03
Correlation with neutrophil count	-0.02*	0.16	0.06*	-0.16	0.07	0.16	0.10	-0.04*	0.10
Correlation with MDSC count	-0.05	-0.18	-0.10	0.03	-0.22	0.11	0.06	-0.30	-0.29
Correlation with $CD8^+$ T cell count	-0.09	-0.02	-0.04	-0.08	0.21	0.21	-0.08	0.39	0.09
Correlation with NK cell count	-0.20	-0.45*	-0.035*	-0.22	0.04	-0.03	-0.28*	-0.02	0.04
Correlation with conventional (myeloid) DC count	0.21	0.40	0.33	0.09	0.15	0.25	0.19	0.36	0.17
Correlation with plasmacytoid DC count	-0.02	0.22	0.14	-0.07	-0.06	0.05	-0.08	0.03	-0.23
Correlation with endothelial cell count (marker of angiogenesis)	-0.24	0.02	-0.18	0.08	-0.09	-0.16	-0.20	0.12	0.09
Correlation with macrophage count	0.13	0.37*	0.27	0.20	0.01	0.07	0.13	0.36	0.10
Correlation with macrophage M1 count	0.02	0.14	0.06	0.08	0.05	-0.04	-0.10	0.33	0.18
Correlation with macrophage M2 count	-0.23	0.00	-0.03	-0.11	-0.12	-0.06	-0.12	0.04	-0.10

↑, red background - expression higher in tumor than in healthy tissue; ↓, blue background - expression lower in tumor than in healthy tissue; ↓, red background - worse prognosis; ↑, blue background – better prognosis; red background - positively correlated with the number of cells; blue background - negatively correlated with the number of cells.
